# Stillbirths: how should its rate be reported, its disability-adjusted-life-years (DALY), and stillbirths adjusted life expectancy

**DOI:** 10.1186/s12911-019-0850-8

**Published:** 2019-07-16

**Authors:** Chander Kant

**Affiliations:** 1 Department of Economics, Seton Hall University, 400 South Orange Avenue, South Orange, NJ 07078 USA; 2 Principal, Kant Research, 1 Fielding Road, Short Hills, NJ 07078 USA

**Keywords:** Different measures of stillbirth rates, Years of life lost due to stillbirths, A more complete measure of life expectancy, Importance of stillbirths in poorer countries

## Abstract

**Background:**

A 2016 study standardized the definition of stillbirths. It estimated the rate as a proportion of total births. A 2015 paper addressed the problem of disability-adjusted life-years (DALY) for stillbirths. There has been no adjustment of life expectancy at birth to account for stillbirths.

**Methods and results:**

We follow mathematical and computational methods, use algebra to derive relationships, and large databases. We express the rate as a proportion of live births and use this rate to adjust life expectancy at birth for stillbirths. We then use the difference between the traditional life expectancy and stillbirths adjusted life expectancy (SALE) to obtain DALY for stillbirths for 194 countries, the Millennium Development Goal regions, and income groups.

We show defining stillbirths’ rate as a proportion of live births enhances stillbirths’ importance, especially in poorer countries; negates some of its under-statement vis-a-vis neonatal mortality rate, accentuates its decrease; and permits inference about relative magnitudes of stillbirths and neonatal mortality from the two rates. Using it, we derive stillbirths adjusted life expectancy, and suggest it reflects a more complete and accurate measure of comparative life expectancies of different countries. Its difference from the traditional life expectancy is used to measure DALY for stillbirths that totals 165.3 million years worldwide.

**Conclusion:**

Stillbirths almost equals neonatal mortality yet have not received almost equal attention. We hope highlighting them and adjusting life expectancy for it will spur health interventions so that grand convergence of health outcomes in different countries can be more rapidly achieved. We also believe SALE is a more complete and accurate measure of comparative life expectancies.

**Electronic supplementary material:**

The online version of this article (10.1186/s12911-019-0850-8) contains supplementary material, which is available to authorized users.

## Background

Health professionals, social scientists, and international organizations have not given as much attention to stillbirths as to neonatal mortality. The first data-set for stillbirths in almost all countries became available in 2006, and was for 2000, while that on neonatal mortality have been available since 1990 [1, 2]. In 2011 stillbirth’s data for 2008 and 2009 became available; and were retrospectively estimated to 1995 for about 40% of the sample [3]. The first international goal on stillbirths (and neonatal mortality) was adopted in 2014 [4]. Using revised and updated estimates, Blencowe and colleagues estimate 2.60 million stillbirths occur yearly [5].

The stillborn rate arguably reflects a country’s quality of health care system to a greater extent than life expectancy (since the latter is affected more by smoking, diet, exercise, public sanitation and pollution) and can be an independent/supplementary health indicator. Causes of stillbirths are not fully understood. In the US, about one-fourth of stillbirths are unexplained; and stillbirths after 24 weeks of pregnancy are primarily due to pregnancy/birth related causes like placenta/ umbilical cord problems, birth defects, and infection [6]. In low income countries, where about 98% of the stillbirths globally occur and almost half of the deliveries take place at home, difficult, prolonged and obstructed labor, infections without adequate treatment, and lack of trained obstetric care (compounded by 35–45% absenteeism of health and extension workers) are the primary causes [7, 8, 9].

Earlier studies define stillbirth as fetal death in third trimester with birthweight of 1000 g or more [1, 3]. When birthweight is unavailable, 28 or more completed weeks of gestation is used (or a length of ≥35 cm if the reported gestation age is not judged reliable). Blencowe and colleagues find using birthweight as the primary criterion reduces number of stillbirths in rich countries by 15%, since fetal growth restriction causes many stillbirths [5]. They use fetal death at 28 or more completed weeks of gestation as their exclusive definition. In poor countries, famine increases stillbirths and fortifying pregnant women’s diet with protein-rich supplements reduces stillbirths by as much as 38% [10, 11, 12]. If mothers’ under-nourishment during pregnancy causes stillbirths, using birthweight lower than 1000 g as the primary criterion will undercount stillbirths in poor countries also. Accordingly, we follow fetal death at 28 or more completed gestation-weeks as our exclusive definition. Using it also excludes voluntary abortion from stillbirths, and protects women’s choice to terminate their pregnancies, since abortion after 28 weeks is rare and is mostly due to severe fetal abnormality or if pregnancy is threatening mother’s life [13, 14].

In addition, “[I] n terms of ethics the ethical concept of the fetus as a patient should replace the discourse of “unborn child” when that phrase is used normatively.” [15] “In term of science it is well recognized that between 20 and 24 weeks, it is likely that the fetus “experiences” touch and pain.” [16] “The inability to communicate does not mean that there is no pain or need of treatment.” [17] Although viability in Western countries is between 22 and 24 gestational weeks, it is higher in low income countries; and 28 weeks is chosen as a pragmatic cut-off limit to classify stillbirths.

The World Health Organization (WHO) notes the widespread perception that stillbirths are unavoidable due to congenital abnormalities [1]. It finds it to be untrue - estimating only 7.4% of stillbirths after 28 weeks are due to such factors. Its wide variation among countries (as shown following) also belies the perception of congenital abnormalities being the primarily cause.

The aim and purpose of this paper is to i) highlight the stillbirth rate that is defined consistent with neonatal mortality rate, ii) use it to adjust life expectancy at births to account for stillbirths, and iii) use the latter to obtain DALY for stillbirths. As will be clear below, the paper is a kind of review proposing new aspects for classification.

### A. Methods: stillbirth rate defined consistent with neonatal mortality rate

There is no consistency among various authors on how they define the stillbirth rate. Some report it (like neonatal mortality) as a proportion of live births [18]. Others, while noting its definition varies among countries and even among states of the US, define it as a proportion of total (= still + live) births [19].

Blencowe and colleagues estimate stillbirth rates based on 2207 data points [5]. They do not break-up data into whether it reported stillbirth rate as a proportion of total or of live births. Since it is natural to define all rates (stillbirths, neonatal, infant, and child mortality) included in a study similarly, it is unlikely all 2207 data points would report stillbirth rate as a proportion of total births. Their definition is apparently based ultimately on Goldenberg and colleagues [20, 21]. Goldenberg and colleagues summarize key findings in the previous five reports in Lancet’s 2011 Stillbirth Series [22, 23, 24, 25, 26]. They define stillbirth rate as “per 1000 births,” not as “per 1000 total births,” at eight places, including in their Conclusion and Call to Action. Since the commonly accepted meaning of “births” is “live births,” by “per 1000 births,” they must mean “per 1000 live births.”

International statistical classification of diseases terms stillbirths (*SB*) as a proportion of live births (*LB*) “fetal death ratio;” and calls stillbirths as a proportion of total births (*TB* = *SB* + *LB*) “fetal death rate.” [27] It encourages both to be reported and requires the denominator to be always specified. Specifying the denominator in the definition itself, we term the two as still live birth rate (*SLBR = SB/LB*) and still total birth rate (*STBR = SB/TB*), respectively.

Using our nomenclature, Blencowe and colleagues provide data for *STBR* [5]. Mortality after live birth with 22 to 27 weeks and 6 days’ gestational age are included in the neonatal mortality rates (*NMR = NM/LB*) while fetal deaths with the same gestational age are excluded from *STBR -* since it includes stillbirths only after 28 weeks gestational age. That understates stillbirth rate’s magnitude vis-à-vis *NMR*. Dividing stillbirths by a bigger number (total births) and neonatal mortality by a smaller number (live births) compounds its understatement.

We can show the difference between *SLBR* and *STBR* is.

1 $$ SLBR- STBR= SLBR\times STBR/1000>0 $$

the two rates either both decrease or both increases, and when they decrease, the rate of decline in *SLBR* must be greater than that in *STBR* (see, Additional file 1).

(1) tells us greater the *SLBR,* greater is its excess over *STBR.* For richer countries where the stillbirth rates are low, *SLBR* and *STBR* will be quite close; but for poorer countries where they are high, the excess of *SLBR* over *STBR* will be significant.

To infer about the relative numbers of stillbirths and neonatal mortality from their relative rates requires that both adverse events be divided by the same number. That requirement is met when *SLBR = SB/LB* is used in the stillbirth rate to *NMR* ratio but not when *STBR = SB/TB* is used.

2 $$ \mathrm{Stillbirth}\ \mathrm{rate}\ \mathrm{to}\kern0.5em NMR\kern0.5em \mathrm{ratio}\ \mathrm{when}\kern0.5em SLBR\kern0.5em \mathrm{is}\ \mathrm{used}=\left( SB/ LB\right)\div \left( NM/ LB\right)= SB/ NM $$

while

3 $$ \mathrm{Stillbirth}\ \mathrm{rate}\ \mathrm{to}\kern0.5em NMR\kern0.5em \mathrm{ratio}\ \mathrm{when}\kern0.5em STBR\kern0.5em \mathrm{is}\ \mathrm{used}=\left( SB/ TB\right)\div \left( NM/ LB\right)=\left( SB/ NM\right)\times \left( LB/ TB\right) $$

Since (*LB/TB*) < 1, stillbirth rate to *NMR* ratio when *STBR* is used is smaller than when *SLBR* is used in the ratio instead.

Blencowe and colleagues use *STBR*:*NMR* ratio of less than 0.33 to exclude 156 data points on grounds that a ratio so low is implausible; and use this ratio of greater than 0.5 as one criterion to classify data from national routine information systems as high quality [5]. But, *STBR*:*NMR* ratio does not equal *SB*: *NM,* as Blencowe and colleagues mistakenly imply; *SLBR*: *NMR* does. This distinction needs to be recognized.

### Results of defining stillbirth rate consistent with neonatal mortality rate

Table 1 compares *SLBR* to *STBR* for countries with the 10 highest stillbirth rates in 2015. Results for all countries are given in Additional file 2. They show excess of *SLBR* over *STBR* is approximately two for Pakistan and Nigeria and between one and two for 12 other countries. *SLBR* is higher than 30 for 14 countries (compared to 13 for *STBR*); its decrease is greater than that in *STBR* by about one percentage point for some countries. See, Additional file 2.

**Table 1:** Countries with ten highest stillbirth rates

Country name	SLBR	STBR	SLBR-STBR
Pakistan	45.09	43.15	1.94
Nigeria	44.81	42.89	1.92
Chad	41.58	39.92	1.66
Guinea-Bissau	38.10	36.70	1.40
Niger	38.07	36.67	1.40
Somalia	36.80	35.49	1.31
Djibouti	35.85	34.61	1.24
Central African Republic	35.59	34.37	1.22
Togo	35.36	34.15	1.21
Mali	33.63	32.53	1.10

SLBR and STBR stand for stillbirth rates defined with respect for live births and total (still + live) births, respectively; and are for 2015 above. Data for STBR is from Blencowe et al. [5]. SLBR is derived by using the number stillborn from Blencowe et al. [5] and number of live births as calculated by using the neonatal mortality number and rate from World Development Indicators

Table 2 provides *SLBR* and *SLBR*:*NMR* ratio for 2000 and 2015 by Millennium Development Goal (MDG) regions and two income groupings: 1) high and upper mid income (richer) and 2) lower mid and low income (poorer). It also provides within region/group standard deviation and dispersion measured as standard deviation/mean (i.e., coefficient of variation), because the means are different. Table 2 shows *SLBR* declined for each region, signifying success. The failure is the increase in its dispersion everywhere (except one region). The increased dispersion is not accounted for anywhere in the recent stillbirth study [5].

**Table 2:** SLBR & SLBR: NMR by region & income-group

	SLBR		SLBR: NMR = SB: NM	
Region/ income-group	2000	2015	2000	2015
DR Rate/Ratio	4.51	3.44	83.5	107.0
SD	2.21	1.63	54.7	68.4
SD/Mean	45.3	46.9	50.9	49.1
SA Rate/Ratio	36.76	26.16	81.1	89.7
SD	15.8	12.8	14.9	27.1
SD/Mean	53.7	65.2	18.8	28.2
CCA Rate/Ratio	17.14	12.02	63.3	74.5
SD	4.85	3.53	26.3	45.4
SD/Mean	25.9	27.5	34.1	43.9
EA Rate/Ratio	14.47	7.21	69.7	128.9
SD	7.14	4.73	27.7	31.6
SD/Mean	57.3	62.3	36.3	29.2
LAC Rate/Ratio	11.4	8.28	77.8	89.3
SD	5.64	4.77	37.3	42.3
SD/Mean	42.9	46.2	39.2	39.1
NAME Rate/Ratio	20.28	14.75	94.6	114.1
SD	8.91	6.86	36.9	95.7
SD/Mean	54.9	57.7	33.2	61.0
SEA Rate/Ratio	17.28	12.37	81.6	90.9
SD	7.22	5.31	37.6	42.7
SD/Mean	44.2	41.9	38.1	39.6
SSA Rate/Ratio	36.92	29.50	90.5	103.2
SD	10.2	8.45	19.7	22.8
SD/Mean	32.9	34.7	23.0	24.2
World Rate/Ratio	25.37	18.73	83.1	97.7
SD	12.5	9.86	38.5	58.8
SD/Mean	72.3	75.5	40.3	50.3
H & Up-Mid-Y Rate/Ratio	11.33	7.40	75.1	101.5
SD	6.43	5.03	46.5	70.9
SD/Mean	66.4	69.7	45.3	54.4
Low-Mid & L-Y Rate/Ratio	33.97	25.28	84.9	97.0
SD	11.1	9.15	19.5	26.1
SD/Mean	40.2	43.2	22.7	26.7

SLBR stands for stillbirth rates defined with respect for live births. It is derived by using the number stillborn from Blencowe et al. [5] and number of live births as calculated by using the neonatal mortality number and rate from World Development Indicators. SB, NM and NMR stand for the number stillborn, neonatal mortality, and its rate, respectively. The abbreviations for the regions and income-groups are *DR* developed region, *SA* Southern Asia, *CCA* Caucasus and Central Asia, *EA* Eastern Asia, *LA* Latin America & Caribbean, *NAME* North Africa and Middle East, *SEA* South-eastern Asia, *SSA* Sub-Saharan Africa, *H & Up-Mid-Y* high and upper middle income, *Low-Mid & L-Y* lower middle and low income. Rate/ratio are for the whole region or income-group. SD and mean are of individual countries’ rates/ratios in the region or income-group given in Additional file 2. SD/mean is expressed as a % (with % sign not written)

Table 2 shows *SLBR*:*NMR* ratio has increased in every region/income group and shows great variation both among regions and over time. E.g., in 2015, in the Caucasus and Central Asia, there were approximately 75 stillbirths for every 100 neonatal deaths; whereas in Eastern Asia, the corresponding number was approximately 130. Eastern Asia also experienced almost doubling of the *SLBR*:*NMR* ratio from 2000 to 2015. This variation across regions and over time needs further investigation.

Comparing progress by two country-income groups, the absolute reduction in poorer (i.e., lower-middle and lower income) countries’ stillborn rate from 2000 to 2015 (ignoring the negative signs) is 8.69 (= 25.28–33.97) and proportionate/percentage reduction is 25.6% (= 8.69/33.97); while the corresponding numbers for richer (i.e., high and upper-middle income) countries are 3.93 (= 7.40–11.33) and 34.7% (= 3.93/11.33), respectively. That is, the percentage reduction, or the rate of decrease, in poorer countries’ stillborn rate is smaller than that in richer countries.

The *SLBR*:*NMR* ratio for both groups increase; but that for the richer group increases by 25.6 (= 101.5–75.1) and 35.1% (= 25.6/75.1) versus 12.1 (= 97.0–84.9) and 14.3% (= 12.1/84.9) for poorer countries. That is, the percentage increase, or the rate of increase, in this ratio is greater for richer countries than for poorer countries.

Let, g (*SLBR*: *NMR*), g (*SLBR*) and g (*NMR*) represent the rates of change in *SLBR*: *NMR*, *SLBR*, and *NMR,* respectively. Then, we can show that

4 $$ {\displaystyle \begin{array}{l}g\ \left( SLBR: NMR\right)=g\ (SLBR)-g\ (NMR)\kern0.5em \mathrm{or}\\ {}g\ (NMR)=g\ (SLBR)-g\ \left( SLBR: NMR\right)\end{array}} $$

Using the above relationship, we can see the richer countries’ *NMR* also decreases by a greater percentage, disregarding the negative sign, of 69.8 (= − 34.7 - 35.1%), or at a greater rate, than for poorer countries’ 39.9 (= − 25.6 - 14.3%). The richer countries, where the 2000 rates were already much lower, achieve a greater proportionate reduction in both rates.

The Newborn Action Plan notes encouragingly that 11 poorer countries have reduced their *NMR* by more than 40% since 2000 [4]. By 2015, a total of 25 (not 11) such countries had passed the 40% threshold (average reduction 46.7%). At the same time, 49 richer countries had also achieved that feat (average reduction 53.5%). See, Additional file 2. Almost twice as many richer countries have achieved a greater than 40% reduction in their *NMR* than poorer countries; and achieved a greater reduction. The poorer countries have taken on and made some progress in an immense task. Yet, richer countries have made even more of a progress - indicating great scope of progress that is possible for poorer countries.

### B. Methods: adjusting life expectancy to account for stillbirths and using it to obtain DALY for stillbirths

The traditional life expectancy at birth (*LE*), also called life expectancy of live births (*LELB*) here, includes premature births and neonates who may live no longer than an hour or a day. The stillborn can occur either antepartum (before labor or delivery) or intrapartum (during labor or delivery); and are highly sensitive to access to timely high-quality antenatal and intrapartum monitoring and care [5]. 40–45% of stillbirths, namely 1.17 million of 2.60 million total stillbirths, are intrapartum [20, 28]. This number is greater than 1.01 million live newborns who die within the first 24 h (36% of total neonatal deaths) [29]. These deaths occur rapidly, and the first minute after an infant is born—the so-called golden minute—is the crucial window for neonatal resuscitation for the 10 million non-breathing infants born annually [20]. The implications are: i) millions of non-breathing neonates are successfully resuscitated; ii) which death is stillbirth and which is neonatal can be subject to considerable error. These errors are more likely when births take place at home–as is common in rural areas of South Asia and Sub-Saharan Africa (the primary regions where most stillbirths occur) [29].

Variation in classification of neonatal mortality and stillbirths at the local level impacts the reported stillbirths and infant mortality rates [30]. Dearth of females in population cohorts since the late 1930s in China has been ascribed to female losses occurring very early in life [31]. Such female live births are simply not reported or reported as stillborn. The Helping Babies Breathe program in Tanzania reduced stillbirths by 24%; and resuscitation training in six poorer countries, reduced stillbirth rates by 31% [32, 33]. We are not proposing that population, that includes all premature live births, include the stillborn. Nevertheless, millions of stillborn, who, by definition, are after 28 weeks of gestation, are simply ignored in the life expectancy measure. We include stillbirths in vital statistics of life expectancy by adjusting it for stillbirths - calling the result stillbirths adjusted life expectancy, *SALE*.

Life expectancy of 1000 live births is 1000 × *LELB*. Dividing this product by 1000 plus the still live-birth rate, *SLBR*, gives us the life expectancy of total (= still + live) births, *LETB,* or stillbirths adjusted life expectancy, *SALE*.

5 $$ SALE= LETB=\left(1000\times LELB\right)/\left(1000+ SLBR\right) $$

For a country with no stillbirths, *SLBR* is zero and *SALE = LE*. For almost every country, stillbirths are positive, and *SALE < LE*. The difference between the two reflects decrease in life expectancy when stillbirths are also considered. Suppose *LELB* is 71 years, and *SLBR* is 13. Then, (5) would mean dividing 71,000 (life expectancy of 1000 live births) by 1013, rather than by 1000. The resulting number being approximately 70 years, the reduction in life expectancy by 1 year is solely due to dividing 71,000 by 1013; i.e., by including the number of stillbirths per 1000 live births in the denominator.

Now we discuss why stillbirths should be include in DALY, and how we obtain DALY for stillbirths.

DALY, while estimating life years lost due to mortality and morbidity, is also used for prioritizing health care spending [35]. Stillbirths are neither included in it nor in the global tracking mechanism such as the Global Burden of Disease estimates. Part of this reluctance may have been due to lack of reliable data on stillbirths in poorer and middle-income countries. Data on its cousin, neonatal mortality, for almost all countries has been available since 1990, while similar data for stillbirths became available only in 2006 [1, 2]. The protein-supplemental study cited above found it decreased low-weight live births by 32% also (in addition to reducing stillbirths by 38%) [12]. If stillbirths are included in DALY, nutrition and medical interventions focused on pregnant mothers may yield benefits in potential DALY reduction that are two to 10 times, and potential cost per DALY reduction one-half to one-tenth [36]. Since DALY is an important population health measure, not counting stillbirth’s reduction in DALY estimates will also yield anomalous situations where a population with a neonatal mortality reduction, whether or not achieved by moving prenatal care resources to post-natal, is considered healthier even if its incidence of late-gestation stillbirths increases.

Other substantive arguments for including stillbirths in DALY estimates are as follows. The current practice violates one of the four general principles underlying DALYs, namely “treating like outcomes as like.” [34] A 28 gestational-age fetus that is stillborn and one that dies 10 minutes after live birth are essentially like outcomes. Yet, the former is not included in DALY estimates while the latter is. We have discussed above how following fetal death at 28 or more completed gestation-weeks as our exclusive definition of stillbirths protects women’s rights and choice to terminate their pregnancies. Including stillbirths in DALY estimates will spur interventions to reduce it - interventions that predominantly focus on pregnant mother’s health, wellbeing, and prenatal and partum care - and will enhance women’s rights and condition [37].

Normally, DALY for premature mortality, or Years of Life Lost (YLL) due to premature mortality in the population, corresponds to the number of deaths multiplied by the life expectancy at the age at which death occurs [38]. This method cannot be used for stillbirths since life expectancy of stillbirths is zero. Therefore, we obtain DALY of stillbirths by multiplying decrease in life expectancy when stillbirths are also considered by the number of live births. That is,

6 $$ \mathrm{DALY}\ \mathrm{or}\ \mathrm{YLL}\ \mathrm{of}\ \mathrm{stillbirths}= LE- SALE=\mid SALE- LEI\mid \times LB $$

Additional file 1 shows the following:

7 $$ \mid SALE- LE\mid = SLBR\times LE/\left(1000+ SLBR\right)>0 $$

and greater is the *SLBR* and/or greater is the *LE*, greater is *LE*’s excess over *SALE.* Both factors in (7) are important: a) Greater the stillbirth rate, more life-years are lost due to stillbirths; b) greater the life expectancy (of live births), the more life-years are lost because a birth is still rather than live.

A recent study (as far as I know the only paper so far suggesting how DALY for stillbirths should be estimated), implicitly assuming life expectancy of a still birth equals that of a live birth, has suggested “the disvalue attached to a fetal death should gradually increase from zero, at 28 weeks gestational age, to a value equaling that of the death of a [fully developed] newborn infant, at the time of birth,” [36] That is, DALY of stillbirths should be zero, or let us say 0.01, at 28 weeks gestational age increasing to 1.00 at full-term; or increasing 100 times. Since a fetus does not develop 100 times from 28 weeks to full-term, this proposal is counter-intuitive and against medical evidence. At 28 weeks gestational age, survival without major morbidity for infants surviving to discharge is closer to one (it is 0.59) than to zero [39]. In a situation where most of the stillbirths (and pre-term neonatal mortality) take place in poor countries where the gestation age at mortality between 28 to 39 weeks are not certain, attempting precision in DALY estimation (which perforce has to make bold assumptions in valuing vastly disparate morbidity) more than in our proposal above will not be productive. In addition, this proposal suffers from its implicit assumption equating life expectancy of a still birth - that is zero - to that of a live birth.

Stillbirths (like neonatal mortality) also cause parental suffering and psychological distress and may affect parents’ life spans. Data for these effects is limited, especially in low income countries [40]. If available, it will be challenging to add it to measure like DALY of stillbirths. Nevertheless, this effect needs to be recognized.

### Results: adjusting life expectancy to account for stillbirths and using it to obtain DALY for stillbirths

Table 3, Panel A summarizes results (from Additional file 3) for 10 countries with largest decrease in life expectancy due to stillbirths. Its Panel B summarizes results for countries with 10 largest DALY of stillbirths that are not included in Panel A. Decrease in life expectancy due to stillbirths is as high as approximately 3 years for Pakistan and approximately 2 (between 1.69 and 2.28) years for 17 other countries. Panel B shows India, with 39.2 million years, has the highest DALY of stillbirths. Its loss exceeds the sum of the next two countries, Nigeria and Pakistan, and is more than four times that in China. Other countries in the top 10 DALY group are Ethiopia, Bangladesh, Congo, Indonesia, Tanzania, and Egypt.

**Table 3:** Countries with highest i) decrease in life expectancy due to stillbirths, and ii) DALY of stillbirths

Country name	LE	SALE	|SALE-LE|	DALY = YLL (in 100,000)
Pakistan	66.38	63.52	2.86	152.4
Nigeria	53.05	50.77	2.28	159.1
Niger	61.97	59.70	2.27	21.6
Djibouti	62.29	60.13	2.16	0.5
Chad	51.87	49.80	2.07	12.5
Togo	60.12	58.07	2.05	5.1
Guinea-Bissau	55.47	53.43	2.04	1.3
Somalia	55.69	53.71	1.98	8.8
Comoros	63.55	61.61	1.94	0.5
Ethiopia	64.58	62.66	1.92	60.3
India	68.35	66.78	1.57	392.0
China	75.99	75.45	0.54	91.8
Bangladesh	72.00	70.17	1.83	58.1
Congo, Dem. Rep.	59.02	57.41	1.61	50.4
Indonesia	69.07	68.16	0.91	50.0
Tanzania	65.49	64.02	1.47	30.1
Egypt, Arab Rep.	71.32	70.45	0.87	24.4

LE and SALE stand for the traditional life expectancy and stillbirths adjusted life expectancy, respectively, and are for 2015 above. Traditional life expectancy data is from World Development Indicators (WDI) Difference between the two measures decrease in life-expectancy due to considering stillbirths. In the first ten rows, countries are ranked by this difference; in the last seven by DALY = YLL (years of life lost) of stillbirths

Table 4 presents results by MDG region and by two country-income groups, richer and poorer. The worldwide mean decreases in life expectancy due to considering stillbirths is 0.85 years. Regions with mean decrease significantly higher and lower than the world average are, for higher: Southern Asia (1.30 years) and Sub-Saharan Africa (1.41 years), and for lower: developed region (0.27 years) and Eastern Asia (0.54 years). The variability of this decrease, measured by standard deviation of the decrease scaled by the mean (since the means are different), among countries in a region is the lowest in the Caucasus and Central Asia (25.8) and Sub-Saharan Africa (30.8), and highest in Southern Asia (60.2) and Eastern Asia (57.9) -suggesting efforts to reduce it may be more successful in the latter two regions. The worldwide DALY = years of lost life due to stillbirths was 165.3 million years in 2015. Of this, 122.3 million (74%) are in Southern Asia and Sub-Saharan Africa. By income, 138 million (83.5%) of DALY due to stillbirths are in poorer countries.

**Table 4:** Stillbirth-caused decrease in life expectancy and DALY or years of life lost by region & income-group

Region/income-group	LE	SALE	|SALE-LE|	DALY=YLL (in 100,000)
DR Mean/Total	79.31	79.04	0.27	3.6
SD	3.7	3.7	0.11	
SD/Mean	4.6	4.7	41.8	
SA Mean/Total	70.53	69.23	1.30	63.4
SD	5.1	5.7	0.79	
SD/Mean	7.2	8.2	60.2	
CCA Mean/Total	70.89	69.99	0.89	1.6
SD	3.1	3.1	0.23	
SD/Mean	4.3	4.5	25.8	
EA Mean/Total	74.58	74.03	0.54	9.7
SD	5.8	6.0	0.32	
SD/Mean	7.7	8.1	57.9	
LAC Mean/Total	74.14	73.40	0.75	6.7
SD	3.8	4.0	0.45	
SD/Mean	5.1	5.4	41.6	
NAME Mean/Tot	74.06	73.21	0.85	10.4
SD	3.7	4.0	0.45	
SD/Mean	5.0	5.5	52.3	
SEA Mean/Total	71.22	70.34	0.88	10.6
SD	4.7	5.0	0.34	
SD/Mean	6.6	7.0	38.9	
SSA Mean/Total	60.33	58.92	1.41	59.4
SD	5.8	6.0	0.43	
SD/Mean	9.7	10.1	30.8	
World Mean/Tot	71.18	70.33	0.85	165.3
SD	8.38	8.82	0.56	
SD/Mean	11.8	12.5	65.2	
H & Up-Mid-Y				
Mean/Total	76.14	75.63	0.51	27.2
SD	5.6	5.9	0.32	
SD/Mean	7.4	7.7	62.5	
Low-Mid & L-Y				
Mean/Total	64.69	63.39	1.30	138.1
SD	6.83	7.07	0.48	
SD/Mean	10.6	11.1	37.0	

LE and SALE stand for the traditional life expectancy and stillbirths adjusted life expectancy, respectively, and are for 2015 above. Traditional life expectancy data is from World Development Indicators (WDI) Difference between the two measures decrease in life-expectancy due to considering stillbirths. SD and mean are of corresponding numbers of individual countries’ in the region or income-group given in Additional file 3. The abbreviations for the regions and income-group are *DR* developed region, *SA* Southern Asia, *CCA* Caucasus and Central Asia, *EA* Eastern Asia, *LA* Latin America & Caribbean, *NAME* North Africa and Middle East, *SEA* South-eastern Asia, *SSA* Sub-Saharan Africa, *H & Up-Mid-Y* high and upper middle income, *Low-Mid & L-Y* lower middle and low income. SD/mean is expressed as a % (with % sign not written)

The gap between average life expectancies between the developed region (rich) and Sub Saharan Africa (poor) is 18.98 years (= 79.31–60.33) for *LE;* and 20.12 years for *SALE* (= 79.04–58.92). We can describe the health convergence objective in two alternative ways: i) poor countries need to increase their life expectancy of live births by approximately 19 years and decrease their still live-birth rate from 29.50 to 3.44 (see, Table 2); or ii) they need to increase their stillbirths adjusted life expectancy by approximately 20.1 years. In some sense, the second may be preferred since it directly incorporates the stillbirth objective in the life expectancy measure. Because many neonatal deaths, 40% of which occur on the first day of life, are misreported as stillbirths, incorporating stillbirths may also reflect a more accurate (and complete) measure of life expectancy.

## Conclusion

### Main findings

Stillbirths almost equals neonatal mortality yet have not received almost equal attention. Defining stillbirths’ rate as a proportion of live births enhances stillbirths’ importance, especially in poorer countries; and negates some of its under-statement vis-a-vis neonatal mortality rate. We employ this definition to adjust life expectancy for stillbirths; and propose the latter to get stillbirths’ DALY that equal 165.3 million years.

### Meaning of the findings, research implications

Stillbirth rate arguably reflects a country’s quality of health care system to a greater extent than life expectancy; and stillbirths adjusted life expectancy reflects a more complete and accurate measure of comparative life expectancies. Including it in DALY will lead to better priorities in health care spending. Highlighting stillbirths and adjusting life expectancy for it will spur research on stillbirths whose causes are not well understood.

### Clinical and health implications

Some key interventions such as syphilis treatment in pregnancy, fetal heart monitoring, and labor surveillance could be crucial in preventing intrapartum stillbirths [3]. In low income countries, only a minority of deliveries occur in health facilities or with the assistance of a trained personnel. This is due to both inadequate resources and absenteeism of health workers [9, 10]. The implication is need for both more resources and better governance. Further, resuscitation training of health care workers in poorer countries is sorely needed.

### Strength and weaknesses

The paper’s strengths are a) highlighting that having different divisors for stillbirths and neonatal mortality rates may give inconsistent results. and b) traditional life expectancy suffers from the limitation that what is stillbirth and what is a live birth is subject to considerable error. Its weakness is that data on stillbirths are not estimated by UN Inter-agency Group for Child Mortality Estimation, childmortality.org. If the latter estimates stillbirths while appraising its neonatal mortality numbers, the estimates of both are likely to improve.

In 2016, the definition of stillbirths was standardized to mean fetal loss after 28 weeks of gestation [5]. It defined stillbirth rate as a proportion of total (still + live) births. We are proposing it be called still total birth rate, and what international statistical classification of diseases terms “fetal death ratio,” (stillbirths as a proportion of live births) be called still live birth rate [27]. The latter accentuates its decline and makes the stillbirth rate comparable to NMR. Using it, we derive stillbirths adjusted life expectancy. Its difference from the traditional life expectancy reflects DALY for stillbirths that totals 165.3 million years worldwide.

There has been a call for better prenatal, natal, and neonatal health monitoring and improved data definitions/methods with consistent metrics [21]. There are triple benefits from such attention: benefits i) for stillbirths, ii) for neonatal deaths, and iii) for mothers; life complications and disability may also be reduced [21]. Stillbirths in poorer countries are another dimension of health that needs to be addressed when seeking, hopefully rapid, grand convergence to health outcomes in richer countries. A stillbirth-incorporated definition of the widely used life expectancy measure will attract more attention to stillbirth. Life expectancy at birth ignores morbidity and is a “very imperfect measure of health.” [41] Adjusting it for stillbirths will also remove some of its imperfections.

## Additional files

Additional file 1: Mathematical derivations; format. (PDF 428 kb)

Additional file 2: Still live-birth and total-birth rates, and SLBR:NMR ratio, 2000 and 2015, by country; format. (PDF 575 kb)

Additional file 3: Life expectancy & stillbirths adjusted life expectancy, 2000 & 2015, and i) decrease in life expectancy due to stillbirths, and ii) DALY = YLL of stillbirths, 2015, by country; format. (PDF 451 kb)

## Data Availability

The datasets analyzed during the current study are available (and no (administrative) permission is required to use/reuse them) in the. a) Supplementary Material to Blencowe et al. (2016) Blencowe H, Cousens S, Jassir F-B, et al. (2016) National, regional, and worldwide estimates of stillborn rates in 2015, with trends from 2000: a systematic analysis. *Lancet Globl Health* 4: e98–108. https://www.thelancet.com/journals/langlo/article/PIIS2214-109X(15)00275-2/fulltext#seccestitle190. b) World Bank. World Development Indicators. The World Bank. Washington, D.C. https://data.worldbank.org/indicator/SP.DYN.IMRT.IN https://data.worldbank.org/indicator/sp.dyn.le00.in c) UN Inter-agency Group for Child Mortality Estimation (UNICEF, WHO, World Bank, UN DESA Population Division) - for neonatal mortality rate and numbers for Cook Islands. childmortality.org d) U.S. Census Bureau: International Database - for life expectancy numbers for Marshall Islands and Palau. https://www.census.gov/programs-surveys/international-programs/about/idb.html

## Abbreviations

DALY: Disability-adjusted life-years
LB: Number of live births
LE = LELB: Life expectancy of live births
LETB: Life expectancy of total (= still + live) births = SALE
MDG: Millennium development goals
NM: Number of neonatal mortalities
NMR: Neonatal mortality rate
SALE: Stillbirths adjusted life expectancy
SB: Number of still births
SLBR: Still live birth rate (= SB/LB)
STBR: Still total (= still + live) birth rate (= SB/TB)
TB: Number of total (= still + live) births
YLL: Years of life lost
