# Supplementary material for: Stillbirths: how should its rate be reported, its disability-adjusted-life-years (DALY), and stillbirths adjusted life expectancy
Source: BMC Med Inform Decis Mak. 2019 Jul 16;19:133. doi: 10.1186/s12911-019-0850-8 (PMC6631739; doi:10.1186/s12911-019-0850-8)
Supplement: Supplementary file 1 — Mathematical derivations; format. (PDF 428 kb) [file 12911_2019_850_MOESM1_ESM.pdf]

Additional file 1: Mathematical derivations

The equations for the two stillbirth rates are:

$STBR$ = SB × 1000/(LB + SB) (A1)

$SLBR$ = SB × 1000/LB (A2)

Multiplying and dividing the right-hand side of (A1) by LB, we have

$STBR = SB \times 1000 \times LB/(LB + SB) \times LB$, or

$STBR = SLBR \times LB/(LB + SB)$ (A1́)

Subtracting the RHS of (A1́), that equals $STBR$, from $SLBR$, we get

$SLBR - STBR = SLBR - [SLBR \times LB/(LB + SB)]$, or

$SLBR - STBR = SLBR\ [1 - LB/(LB + SB)]$, or

$SLBR - STBR = SLBR\ [(LB + SB) - LB]/(LB + SB)]$, or

$SLBR - STBR = SLBR \times SB/(LB + SB) = SLBR \times STBR/1000 > 0$ (A3)

We now derive expressions for the changes in the two stillbirth rates. Let $SLBR_1$, $STBR_1$, $SLBR_2$, and $STBR_2$ represent the two stillbirth rates, and LB_1, SB_1, LB_2, and SB_2 the number of live and stillbirths for years 1 and 2, respectively. Let

$SB_2 = kSB_1$, where $k > 0$. (A4).

When $k > 1$, the number of stillbirths in year 2 is greater than that in year 1; when it is < 1, the number in year 2 is smaller.

Let $\Delta SLBR$ and $\Delta STBR$ denote the change in the two rates from year 1 to year 2. Then,

$\Delta SLBR = (1000 \times kSB_1/LB_2) - (1000 \times SB_1/LB_1)$, or

$\Delta SLBR = (1000 \times SB_1)\ (k/LB_2 - 1/LB_1)$, or

$\Delta SLBR = (1000 \times SB_1) \times (kLB_1 - LB_2)/\ (LB_2 \times LB_1)$ (A5),

and

$\Delta STBR = (1000 \times kSB_1/(LB_2 + kSB_1)) - (1000 \times SB_1/(LB_1 + SB_1))$, or

$\Delta STBR = (1000 \times SB_1)\ ((k/(LB_2 + kSB_1) - 1(/LB_1 + SB_1))$, or

$\Delta STBR = (1000 \times SB_1)\ (((k \times (LB_1 + SB_1) - (LB_2 + kSB_1))/\ (LB_2 + kSB_1)\ (LB_1 + SB_1))$, or

$$\Delta STBR = (1000 \times SB_1)\ ((kLB_1 - LB_2)\ /(LB_2 + kSB_1)\ (LB_1 + SB_1)) \qquad \text{(A6).}$$

Examining the right hand sides of (A5) and (A6), we get

$$\text{sign } \Delta SLBR = \text{sign } \Delta STBR = \text{sign } (kLB_1 - LB_2) \qquad \text{(A7).}$$

Thus, the two rates change in the same direction: They either both decrease or they both increase.

The proportionate (i.e., ignoring the 100) rate of change (= growth or reduction) in the two stillbirth rates are

$$\Delta SLBR/\ SLBR_1 = (1000 \times SB_1) \times (kLB_1 - LB_2)/\ (LB_2 \times LB_1) \div (1000 \times SB_1/LB_1), \text{ or}$$

$$\Delta SLBR/\ SLBR_1 = (kLB_1 - LB_2)/\ LB_2 \qquad \text{(A8)}$$

and

$$\Delta STBR/\ STBR_1 = (1000 \times SB_1)\ ((kLB_1 - LB_2)\ /(LB_2 + kSB_1)\ (LB_1 + SB_1)) \div (1000 \times SB_1/(LB_1 + SB_1)$$

or

$$\Delta STBR/\ STBR_1 = ((kLB_1 - LB_2)\ /(LB_2 + kSB_1) \qquad \text{(A9)}$$

Dividing (A8) by (A9), we have

$$[(\Delta SLBR/\ SLBR_1)/(\Delta STBR/\ STBR_1)] = [(LB_2 + kSB_1)/LB_2] > 1. \qquad \text{(A10)}$$

(A10) implies the absolute rate of change in SLBR must be greater than that in STBR. When the two rates are falling, it means the rate of fall in SLBR must be greater than that in STBR. When they are increasing, the rate of increase in SLBR must be greater than that in STBR.

<u>The two life expectancies:</u>

Subtracting the RHS of (5) in the text from $LE = LELB$, we have

$$LELB - LETB = LELB\ (1 - (1000\ /(1000 + SLBR))), \text{ or}$$

$$LELB - LETB = LE - SALE = S\ LBR \times LE/(1000 + SLBR) > 0. \qquad \text{(A11),}$$

Let ΔLE represent the excess of *LE* over *SALE* (that equals the shortfall of *SALE* from *LE*). The partial derivative of ΔLE with respect to *SLBR* is positive. That and (A11) tell us that greater is the *SLBR* and/or greater is the *LE*, greater is *LE*'s excess over *SALE* (or, greater is *SALE*'s shortfall from *LE).*
