# Supplementary material for: Stillbirths: how should its rate be reported, its disability-adjusted-life-years (DALY), and stillbirths adjusted life expectancy
Source: BMC Med Inform Decis Mak. 2019 Jul 16;19:133. doi: 10.1186/s12911-019-0850-8 (PMC6631739; doi:10.1186/s12911-019-0850-8)
Supplement: Supplementary file 2 — Still live-birth and total-birth rates, and SLBR:NMR ratio, 2000 and 2015, by country; format. (PDF 575 kb) [file 12911_2019_850_MOESM2_ESM.pdf]

Additional file 2: Still live-birth and total-birth rates, and SLBR:NMR ratio, 2000 and 2015, by country

			2000		2015			% Δ 2000-2015				SLBR:NMR=SB:NM	
**Country name**	**MDG region**	**Income group**	**SLBR**	**STBR**	**SLBR**	**STBR**	**SLBR-STBR**	**SLBR**	**STBR**	**|SLBR-STBR|**	**NMR**	**2000**	**2015**
Afghanistan	Southern Asia	Low income	37.01	35.69	27.48	26.75	0.73	-25.75	-25.05	0.70	-21.37	81.6	77.1
Albania	Developed region	Upper middle income	5.79	5.76	4.00	3.98	0.02	-30.92	-30.90	0.02	-45.12	51.4	64.7
Algeria	North Africa and Middle East	Upper middle income	26.70	26.01	19.70	19.32	0.38	-26.22	-25.72	0.50	-26.04	127.6	127.3
Andorra	Developed region	High income:	1.10	1.10	1.40	1.40	0.00	27.27	27.27	0.00	-59.62	42.3	133.3
Angola	Sub-Saharan Africa	Upper middle income	35.49	34.27	28.08	27.31	0.77	-20.88	-20.31	0.57	-16.46	60.6	57.4
Antigua and Barbuda	Latin America and the Caribbean	High income:	8.77	8.70	7.00	6.95	0.05	-20.18	-20.11	0.07	-52.07	93.2	155.2
Argentina	Latin America and the Caribbean	High income:	7.37	7.32	4.60	4.57	0.03	-37.58	-37.57	0.01	-44.24	65.3	73.1
Armenia	Caucasus and Central Asia	Lower middle income	21.48	21.03	13.96	13.77	0.19	-35.01	-34.52	0.49	-53.40	135.4	188.9
Australia	Developed region	High income: OECD	3.37	3.36	2.72	2.71	0.01	-19.29	-19.35	-0.06	-37.14	96.3	123.6
Austria	Developed region	High income: OECD	4.67	4.65	3.64	3.63	0.01	-22.06	-21.94	0.12	-32.26	150.6	173.3
Azerbaijan	Caucasus and Central Asia	Upper middle income	25.85	25.20	16.74	16.47	0.27	-35.24	-34.64	0.60	-45.56	77.1	91.7
Bahamas, The	Latin America and the Caribbean	High income:	11.27	11.15	10.35	10.24	0.11	-8.16	-8.16	0.00	-19.84	130	148.9
Bahrain	North Africa and Middle East	High income:	9.48	9.39	5.55	5.52	0.03	-41.46	-41.21	0.25	-76.43	201.3	500.0
Bangladesh	Southern Asia	Lower middle income	44.22	42.35	26.02	25.36	0.66	-41.16	-40.12	1.04	-45.47	103	111.1
Barbados	Latin America and the Caribbean	High income:	9.28	9.20	8.59	8.52	0.07	-7.44	-7.39	0.05	-7.49	108.7	108.7
Belarus	Developed region	Upper middle income	5.66	5.63	2.97	2.96	0.01	-47.53	-47.42	0.11	-74.29	76.6	156.3
Belgium	Developed region	High income: OECD	3.55	3.54	3.02	3.01	0.01	-14.93	-14.97	-0.04	-26.67	118.3	137.3
Belize	Latin America and the Caribbean	Upper middle income	11.29	11.16	9.84	9.75	0.09	-12.84	-12.63	0.21	-33.76	90.3	118.8
Benin	Sub-Saharan Africa	Low income	37.46	36.11	31.24	30.30	0.94	-16.6	-16.09	0.51	-19.59	94.5	98.0
Bhutan	Southern Asia	Lower middle income	27.59	26.85	16.19	15.94	0.25	-41.32	-40.63	0.69	-44.16	84	88.2
Bolivia	Latin America and the Caribbean	Lower middle income	18.06	17.74	13.04	12.87	0.17	-27.8	-27.45	0.35	-34.41	60.2	66.3
Bosnia and Herzegovina	Developed region	Upper middle income	6.74	6.70	5.48	5.45	0.03	-18.69	-18.66	0.03	-39.70	102.1	137.7
Botswana	Sub-Saharan Africa	Upper middle income	17.84	17.53	15.46	15.22	0.24	-13.34	-13.18	0.16	-8.49	74.2	70.3
Brazil	Latin America and the Caribbean	Upper middle income	12.24	12.09	8.65	8.58	0.07	-29.33	-29.03	0.30	-44.23	76.8	97.3
Brunei Darussalam	Southeastern Asia and Oceania	High income:	6.98	6.93	6.52	6.47	0.05	-6.59	-6.64	-0.05	-12.60	141.9	151.6
Bulgaria	Developed region	Upper middle income	8.54	8.46	5.77	5.73	0.04	-32.44	-32.27	0.17	-50.44	75.7	103.2
Burkina Faso	Sub-Saharan Africa	Low income	30.25	29.36	21.70	21.24	0.46	-28.26	-27.66	0.60	-37.07	71.2	81.2
Burundi	Sub-Saharan Africa	Low income	37.27	35.93	27.36	26.63	0.73	-26.59	-25.88	0.71	-27.22	94.5	95.4
Cabo Verde	Sub-Saharan Africa	Lower middle income	19.76	19.38	14.49	14.29	0.20	-26.67	-26.26	0.41	-29.55	114.3	119.0
Cambodia	Southeastern Asia and Oceania	Low income	21.27	20.83	12.08	11.94	0.14	-43.21	-42.68	0.53	-58.99	58.8	81.5
Cameroon	Sub-Saharan Africa	Lower middle income	24.70	24.10	19.96	19.57	0.39	-19.19	-18.80	0.39	-25.74	71.3	77.6
Canada	Developed region	High income: OECD	3.54	3.53	3.15	3.14	0.01	-11.02	-11.05	-0.03	-13.51	95.7	98.4
Central African Republic	Sub-Saharan Africa	Low income	38.80	37.35	35.59	34.37	1.22	-8.27	-7.98	0.29	-12.94	79.1	83.3
Chad	Sub-Saharan Africa	Low income	43.19	41.40	41.58	39.92	1.66	-3.73	-3.57	0.16	-15.80	92.3	105.6
Chile	Latin America and the Caribbean	High income: OECD	3.91	3.89	3.07	3.06	0.01	-21.48	-21.34	0.14	-14.21	68.6	62.8
China	Eastern Asia	Upper middle income	14.73	14.52	7.20	7.15	0.05	-51.12	-50.76	0.36	-74.05	69.6	131.1
Colombia	Latin America and the Caribbean	Upper middle income	11.00	10.88	8.14	8.08	0.06	-26	-25.74	0.26	-36.97	81.7	95.9
Comoros	Sub-Saharan Africa	Low income	35.69	34.46	31.51	30.55	0.96	-11.71	-11.35	0.36	-18.70	84.9	92.2
Congo, Dem. Rep.	Sub-Saharan Africa	Low income	35.40	34.19	28.03	27.27	0.76	-20.82	-20.24	0.58	-22.03	91.7	93.2
Congo, Rep.	Sub-Saharan Africa	Lower middle income	21.49	21.04	15.29	15.06	0.23	-28.85	-28.42	0.43	-45.71	64.7	84.8
Cook Islands	Southeastern Asia and Oceania	Upper middle income	11.36	11.24	6.59	6.55	0.04	-41.99	-41.73	0.26	-28.05	118.5	95.5
Costa Rica	Latin America and the Caribbean	Upper middle income	6.14	6.10	6.02	5.99	0.03	-1.95	-1.80	0.15	-19.38	79.8	97.1
Cote d'Ivoire	Sub-Saharan Africa	Lower middle income	32.87	31.82	27.42	26.69	0.73	-16.58	-16.12	0.46	-23.99	65.6	71.9
Croatia	Developed region	High income:	3.61	3.60	1.99	1.99	0.00	-44.88	-44.72	0.16	-52.46	65.8	76.2
Cuba	Latin America and the Caribbean	Upper middle income	11.11	10.99	6.18	6.15	0.03	-44.37	-44.04	0.33	-45.24	264.5	268.7

Cyprus	Developed region	High income:	5.12	5.09	3.71	3.70	0.01	-27.54	-27.31	0.23	-54.32	158	250.7
Czech Republic	Developed region	High income: OECD	3.31	3.30	2.51	2.50	0.01	-24.17	-24.24	-0.07	-53.85	84.9	139.4
Denmark	Developed region	High income: OECD	3.42	3.41	1.74	1.74	0.00	-49.12	-48.97	0.15	-29.06	97.4	69.9
Djibouti	Sub-Saharan Africa	Lower middle income	45.71	43.71	35.85	34.61	1.24	-21.57	-20.82	0.75	-24.36	103	106.8
Dominica	Latin America and the Caribbean	Upper middle income	10.27	10.17	11.93	11.79	0.14	16.16	15.93	-0.23	38.24	89.5	75.2
Dominican Republic	Latin America and the Caribbean	Upper middle income	13.49	13.31	11.19	11.06	0.13	-17.05	-16.90	0.15	-9.77	56.1	51.5
Ecuador	Latin America and the Caribbean	Upper middle income	11.03	10.91	7.74	7.68	0.06	-29.83	-29.61	0.22	-37.51	63.9	71.8
Egypt, Arab Rep.	North Africa and Middle East	Lower middle income	18.31	17.99	12.38	12.23	0.15	-32.39	-32.02	0.37	-42.46	82.4	96.9
El Salvador	Latin America and the Caribbean	Lower middle income	17.61	17.31	12.30	12.15	0.15	-30.15	-29.81	0.34	-42.26	122.8	148.6
Equatorial Guinea	Sub-Saharan Africa	High income:	21.62	21.16	16.44	16.18	0.26	-23.96	-23.53	0.43	-26.00	48.2	49.5
Eritrea	Sub-Saharan Africa	Low income	28.59	27.80	23.04	22.52	0.52	-19.41	-18.99	0.42	-29.97	108.6	124.9
Estonia	Developed region	High income: OECD	4.25	4.24	2.67	2.66	0.01	-37.18	-37.26	-0.08	-76.40	67.8	180.4
Ethiopia	Sub-Saharan Africa	Low income	40.70	39.11	30.59	29.68	0.91	-24.84	-24.11	0.73	-42.80	83.6	109.8
Fiji	Southeastern Asia and Oceania	Upper middle income	14.36	14.16	12.06	11.92	0.14	-16.02	-15.82	0.20	-31.05	103.7	126.3
Finland	Developed region	High income: OECD	2.65	2.65	1.85	1.85	0.00	-30.19	-30.19	0.00	-48.00	106	142.3
France	Developed region	High income: OECD	5.49	5.46	4.74	4.72	0.02	-13.66	-13.55	0.11	-21.43	196.1	215.5
Gabon	Sub-Saharan Africa	Upper middle income	17.15	16.86	14.15	13.96	0.19	-17.49	-17.20	0.29	-22.13	57.3	60.8
Gambia, The	Sub-Saharan Africa	Low income	31.72	30.74	24.44	23.86	0.58	-22.95	-22.38	0.57	-28.50	75.5	81.3
Georgia	Caucasus and Central Asia	Lower middle income	18.95	18.59	11.36	11.24	0.12	-40.05	-39.54	0.51	-65.65	90.4	157.8
Germany	Developed region	High income: OECD	2.67	2.66	2.43	2.43	0.00	-8.99	-8.65	0.34	-25.00	95.4	115.7
Ghana	Sub-Saharan Africa	Lower middle income	30.56	29.65	23.22	22.69	0.53	-24.02	-23.47	0.55	-22.23	83.5	81.6
Greece	Developed region	High income: OECD	4.74	4.71	3.57	3.56	0.01	-24.68	-24.42	0.26	-48.39	84.6	123.5
Grenada	Latin America and the Caribbean	Upper middle income	9.06	8.98	8.00	7.94	0.06	-11.7	-11.58	0.12	-19.97	117.5	129.7
Guatemala	Latin America and the Caribbean	Lower middle income	17.16	16.87	12.08	11.94	0.14	-29.6	-29.22	0.38	-35.98	82.2	90.4
Guinea	Sub-Saharan Africa	Low income	27.83	27.08	21.59	21.13	0.46	-22.42	-21.97	0.45	-35.19	57.4	68.8
Guinea-Bissau	Sub-Saharan Africa	Low income	55.43	52.52	38.10	36.70	1.40	-31.26	-30.12	1.14	-28.65	98.9	95.3
Guyana	Latin America and the Caribbean	Lower middle income	20.13	19.74	17.55	17.25	0.30	-12.82	-12.61	0.21	-12.20	77	76.4
Haiti	Latin America and the Caribbean	Low income	29.43	28.59	25.56	24.92	0.64	-13.15	-12.84	0.31	-16.83	96.2	100.4
Honduras	Latin America and the Caribbean	Lower middle income	18.06	17.74	12.77	12.61	0.16	-29.29	-28.92	0.37	-37.40	103	116.3
Hungary	Developed region	High income: OECD	4.19	4.18	3.68	3.67	0.01	-12.17	-12.20	-0.03	-50.00	59.9	105.1
Iceland	Developed region	High income: OECD	2.89	2.88	1.13	1.12	0.01	-60.9	-61.11	-0.21	-56.04	139.6	124.2
India	Southern Asia	Lower middle income	34.46	33.31	23.57	23.03	0.54	-31.6	-30.86	0.74	-38.76	75.7	84.6
Indonesia	Southeastern Asia and Oceania	Lower middle income	17.99	17.67	13.41	13.23	0.18	-25.46	-25.13	0.33	-39.24	81.1	99.6
Iran, Islamic Rep.	Southern Asia	Upper middle income	9.53	9.44	6.48	6.44	0.04	-32	-31.78	0.22	-50.47	49.7	68.3
Iraq	North Africa and Middle East	Upper middle income	19.99	19.60	15.78	15.54	0.24	-21.06	-20.71	0.35	-24.37	81.9	85.4
Ireland	Developed region	High income: OECD	4.68	4.66	2.73	2.73	0.00	-41.67	-41.42	0.25	-42.36	117.3	118.7
Israel	Developed region	High income: OECD	4.82	4.80	4.21	4.19	0.02	-12.66	-12.71	-0.05	-41.67	133.9	200.5
Italy	Developed region	High income: OECD	3.98	3.96	3.35	3.34	0.01	-15.83	-15.66	0.17	-38.24	117.1	159.5
Jamaica	Latin America and the Caribbean	Upper middle income	21.39	20.95	19.15	18.79	0.36	-10.47	-10.31	0.16	-31.69	125.7	164.8
Japan	Developed region	High income: OECD	3.07	3.06	2.08	2.07	0.01	-32.25	-32.35	-0.10	-50.00	170.6	231.1
Jordan	North Africa and Middle East	Upper middle income	13.14	12.97	10.60	10.48	0.12	-19.33	-19.20	0.13	-36.13	79.3	100.1
Kazakhstan	Caucasus and Central Asia	Upper middle income	11.18	11.06	6.52	6.48	0.04	-41.68	-41.41	0.27	-65.21	55.5	93.0
Kenya	Sub-Saharan Africa	Lower middle income	27.16	26.44	23.02	22.50	0.52	-15.24	-14.90	0.34	-23.48	93.2	103.3
Kiribati	Southeastern Asia and Oceania	Lower middle income	18.88	18.53	16.63	16.35	0.28	-11.92	-11.76	0.16	-18.47	64.8	70.0
Korea, Dem People's Rep	Eastern Asia	Low income	19.80	19.42	13.69	13.50	0.19	-30.86	-30.48	0.38	-50.39	72.9	101.6
Korea, Rep.	Eastern Asia	High income: OECD	2.76	2.75	2.14	2.13	0.01	-22.46	-22.55	-0.09	-33.33	115	133.8
Kuwait	North Africa and Middle East	High income:	6.65	6.61	5.10	5.08	0.02	-23.31	-23.15	0.16	-50.62	102.6	159.4
Kyrgyz Republic	Caucasus and Central Asia	Lower middle income	13.95	13.76	10.33	10.22	0.11	-25.95	-25.73	0.22	-47.00	64.4	90.0

Lao PDR	Southeastern Asia and Oceania	Lower middle income	32.68	31.64	24.30	23.72	0.58	-25.64	-25.03	0.61	-30.50	75.1	80.4
Latvia	Developed region	High income:	5.31	5.28	3.61	3.60	0.01	-32.02	-31.82	0.20	-49.90	51.3	69.6
Lebanon	North Africa and Middle East	Upper middle income	14.14	13.95	9.97	9.87	0.10	-29.49	-29.25	0.24	-59.25	120	207.7
Lesotho	Sub-Saharan Africa	Lower middle income	24.77	24.17	19.91	19.52	0.39	-19.62	-19.24	0.38	-13.19	65.6	60.7
Liberia	Sub-Saharan Africa	Low income	32.51	31.49	21.88	21.42	0.46	-32.7	-31.98	0.72	-44.58	74.7	90.7
Libya	North Africa and Middle East	Upper middle income	12.64	12.49	8.87	8.79	0.08	-29.83	-29.62	0.21	-52.26	83.9	123.4
Lithuania	Developed region	High income:	5.82	5.78	3.24	3.23	0.01	-44.33	-44.12	0.21	-54.55	105.8	129.6
Luxembourg	Developed region	High income: OECD	3.88	3.86	2.85	2.84	0.01	-26.55	-26.42	0.13	-62.66	161	316.7
Macedonia, FYR	Developed region	Upper middle income	10.91	10.79	7.72	7.66	0.06	-29.24	-29.01	0.23	-61.81	118.7	219.9
Madagascar	Sub-Saharan Africa	Low income	21.96	21.49	18.54	18.20	0.34	-15.57	-15.31	0.26	-37.80	69.2	93.9
Malawi	Sub-Saharan Africa	Low income	29.57	28.72	22.30	21.82	0.48	-24.59	-24.03	0.56	-38.77	82.9	102.1
Malaysia	Southeastern Asia and Oceania	Upper middle income	8.05	7.98	5.88	5.85	0.03	-26.96	-26.69	0.27	-26.28	152.2	150.8
Maldives	Southern Asia	Upper middle income	19.56	19.18	7.87	7.80	0.07	-59.76	-59.33	0.43	-81.24	75.7	162.3
Mali	Sub-Saharan Africa	Low income	45.98	43.96	33.63	32.53	1.10	-26.86	-26.00	0.86	-33.53	80.6	88.6
Malta	Developed region	High income:	4.98	4.96	3.58	3.56	0.02	-28.11	-28.23	-0.12	-15.69	97.6	83.3
Marshall Islands	Southeastern Asia and Oceania	Upper middle income	17.60	17.29	15.25	15.02	0.23	-13.35	-13.13	0.22	-7.05	96.1	89.6
Mauritania	Sub-Saharan Africa	Lower middle income	33.60	32.51	27.82	27.07	0.75	-17.2	-16.73	0.47	-17.11	77.7	77.6
Mauritius	Sub-Saharan Africa	Upper middle income	13.31	13.14	9.60	9.51	0.09	-27.87	-27.63	0.24	-31.63	108.2	114.1
Mexico	Latin America and the Caribbean	Upper middle income	7.03	6.98	5.52	5.49	0.03	-21.48	-21.35	0.13	-30.52	69.9	79.0
Micronesia, Fed. Sts.	Southeastern Asia and Oceania	Lower middle income	21.11	20.68	18.39	18.06	0.33	-12.88	-12.67	0.21	-27.33	82.8	99.2
Moldova	Developed region	Lower middle income	10.44	10.33	7.93	7.87	0.06	-24.04	-23.81	0.23	-42.53	50.5	66.7
Monaco	Developed region	High income:	5.60	5.57	5.70	5.67	0.03	1.79	1.80	0.01	-66.67	133.3	407.1
Mongolia	Eastern Asia	Upper middle income	12.58	12.42	7.34	7.29	0.05	-41.65	-41.30	0.35	-56.87	48.8	66.0
Montenegro	Developed region	Upper middle income	6.00	5.96	3.99	3.97	0.02	-33.5	-33.39	0.11	-66.59	66.6	132.6
Morocco	North Africa and Middle East	Lower middle income	34.99	33.81	25.13	24.52	0.61	-28.18	-27.48	0.70	-36.07	126.6	142.2
Mozambique	Sub-Saharan Africa	Low income	27.85	27.09	19.50	19.13	0.37	-29.98	-29.38	0.60	-38.34	63.3	71.8
Myanmar	Southeastern Asia and Oceania	Lower middle income	30.69	29.78	20.36	19.96	0.40	-33.66	-32.98	0.68	-29.33	81.8	76.7
Namibia	Sub-Saharan Africa	Upper middle income	13.42	13.25	11.38	11.25	0.13	-15.2	-15.09	0.11	-18.73	68.5	71.5
Nauru	Southeastern Asia and Oceania	East Asia & Pacific	18.63	18.29	15.15	14.93	0.22	-18.68	-18.37	0.31	-1.44	83.8	69.2
Nepal	Southern Asia	Low income	28.77	27.97	18.72	18.38	0.34	-34.93	-34.29	0.64	-43.66	72.7	83.9
Netherlands	Developed region	High income: OECD	5.31	5.28	1.83	1.82	0.01	-65.54	-65.53	0.01	-36.84	139.7	76.3
New Zealand	Developed region	High income: OECD	3.46	3.45	2.26	2.25	0.01	-34.68	-34.78	-0.10	-11.71	98.9	73.1
Nicaragua	Latin America and the Caribbean	Lower middle income	11.02	10.90	7.50	7.44	0.06	-31.94	-31.74	0.20	-42.90	64.4	76.8
Niger	Sub-Saharan Africa	Low income	40.62	39.04	38.07	36.67	1.40	-6.28	-6.07	0.21	-38.19	93.5	141.8
Nigeria	Sub-Saharan Africa	Lower middle income	55.17	52.29	44.81	42.89	1.92	-18.78	-17.98	0.80	-28.87	114.1	130.3
Norway	Developed region	High income: OECD	3.72	3.71	2.19	2.19	0.00	-41.13	-40.97	0.16	-44.61	138.3	147.0
Oman	North Africa and Middle East	High income:	9.99	9.90	8.51	8.44	0.07	-14.81	-14.75	0.06	-30.67	133.2	163.7
Pakistan	Southern Asia	Lower middle income	56.35	53.34	45.09	43.15	1.94	-19.98	-19.10	0.88	-24.91	92.1	98.2
Palau	Southeastern Asia and Oceania	Upper middle income	10.95	10.83	9.01	8.93	0.08	-17.72	-17.54	0.18	-30.32	71.7	84.7
Panama	Latin America and the Caribbean	Upper middle income	8.77	8.69	6.18	6.14	0.04	-29.53	-29.34	0.19	-34.56	59.8	64.4
Papua New Guinea	Southeastern Asia and Oceania	Lower middle income	19.21	18.85	16.17	15.91	0.26	-15.83	-15.60	0.23	-18.62	63.6	65.8
Paraguay	Latin America and the Caribbean	Upper middle income	19.19	18.83	13.57	13.39	0.18	-29.29	-28.89	0.40	-38.36	108.7	124.7
Peru	Latin America and the Caribbean	Upper middle income	13.92	13.73	9.03	8.95	0.08	-35.13	-34.81	0.32	-47.93	88.5	110.3
Philippines	Southeastern Asia and Oceania	Lower middle income	14.53	14.33	10.99	10.87	0.12	-24.36	-24.15	0.21	-24.97	86.8	87.5
Poland	Developed region	High income: OECD	4.69	4.67	2.35	2.35	0.00	-49.89	-49.68	0.21	-46.55	80.9	75.8
Portugal	Developed region	High income: OECD	3.70	3.69	2.18	2.17	0.01	-41.08	-41.19	-0.11	-41.18	108.8	109.0
Qatar	North Africa and Middle East	High income:	7.58	7.53	5.75	5.72	0.03	-24.14	-24.04	0.10	-41.18	117.3	151.3
Romania	Developed region	Upper middle income	6.80	6.76	4.15	4.13	0.02	-38.97	-38.91	0.06	-51.84	52.1	66.0

Russian Federation	Developed region	High income:	7.75	7.69	4.49	4.47	0.02	-42.06	-41.87	0.19	-59.94	62.1	89.8
Rwanda	Sub-Saharan Africa	Low income	27.82	27.07	17.59	17.29	0.30	-36.77	-36.13	0.64	-55.60	65.8	93.8
Samoa	Southeastern Asia and Oceania	Lower middle income	12.10	11.95	11.05	10.93	0.12	-8.68	-8.54	0.14	-18.26	104.2	116.4
San Marino	Developed region	High income:	4.90	4.88	2.60	2.59	0.01	-46.94	-46.93	0.01	-46.15	376.9	371.4
Sao Tome and Principe	Sub-Saharan Africa	Lower middle income	24.88	24.28	16.47	16.21	0.26	-33.8	-33.24	0.56	-29.71	101.6	95.6
Saudi Arabia	North Africa and Middle East	High income	19.22	18.86	14.13	13.93	0.20	-26.48	-26.14	0.34	-36.27	155.3	179.1
Senegal	Sub-Saharan Africa	Lower middle income	35.89	34.65	25.09	24.47	0.62	-30.09	-29.38	0.71	-46.73	91.7	120.4
Serbia	Developed region	Upper middle income	7.84	7.78	5.98	5.95	0.03	-23.72	-23.52	0.20	-45.45	101.8	142.4
Seychelles	Sub-Saharan Africa	High income	11.32	11.20	9.83	9.73	0.10	-13.16	-13.13	0.03	-12.96	120.3	120.0
Sierra Leone	Sub-Saharan Africa	Low income	40.71	39.12	24.96	24.36	0.60	-38.69	-37.73	0.96	-30.04	81.6	71.6
Singapore	Southeastern Asia and Oceania	High income:	3.53	3.52	2.58	2.58	0.00	-26.91	-26.70	0.21	-38.13	220.6	260.6
Slovak Republic	Developed region	High income: OECD	3.91	3.89	2.94	2.93	0.01	-24.81	-24.68	0.13	-42.68	53.5	70.2
Slovenia	Developed region	High income: OECD	2.70	2.70	2.85	2.84	0.01	5.56	5.19	-0.37	-57.10	81.6	200.7
Solomon Islands	Southeastern Asia and Oceania	Lower middle income	20.04	19.65	17.91	17.59	0.32	-10.63	-10.48	0.15	-15.71	139.3	147.7
Somalia	Sub-Saharan Africa	Low income	40.82	39.22	36.80	35.49	1.31	-9.85	-9.51	0.34	-10.62	91.7	92.5
South Africa	Sub-Saharan Africa	Upper middle income	22.94	22.42	17.76	17.45	0.31	-22.58	-22.17	0.41	-31.16	143.8	161.7
South Sudan	Sub-Saharan Africa	Low income	37.28	35.94	30.98	30.05	0.93	-16.9	-16.39	0.51	-31.60	64.4	78.3
Spain	Developed region	High income: OECD	3.31	3.30	2.88	2.87	0.01	-12.99	-13.03	-0.04	-30.00	82.8	102.9
Sri Lanka	Southern Asia	Lower middle income	7.60	7.54	4.91	4.89	0.02	-35.39	-35.15	0.24	-46.48	75.3	90.9
St. Kitts and Nevis	Latin America and the Caribbean	High income:	9.67	9.58	7.58	7.53	0.05	-21.61	-21.40	0.21	-47.50	80.6	120.3
St. Lucia	Latin America and the Caribbean	Upper middle income	13.18	13.00	12.16	12.01	0.15	-7.74	-7.62	0.12	-18.42	117.3	132.6
St Vincent & Grenadines	Latin America and the Caribbean	Upper middle income	12.57	12.41	10.90	10.78	0.12	-13.29	-13.13	0.16	-13.99	92.6	93.3
Sudan	Sub-Saharan Africa	Lower middle income	30.09	29.21	24.99	24.38	0.61	-16.95	-16.54	0.41	-16.20	84.2	83.4
Suriname	Latin America and the Caribbean	Upper middle income	24.64	24.05	19.82	19.43	0.39	-19.56	-19.21	0.35	-38.26	133	173.3
Swaziland	Sub-Saharan Africa	Lower middle income	16.20	15.94	12.50	12.35	0.15	-22.84	-22.52	0.32	-33.08	76.6	88.3
Sweden	Developed region	High income: OECD	3.81	3.80	2.86	2.85	0.01	-24.93	-25.00	-0.07	-30.13	166.4	178.8
Switzerland	Developed region	High income: OECD	3.25	3.24	2.82	2.81	0.01	-13.23	-13.27	-0.04	-22.86	92.9	104.4
Syrian Arab Republic	North Africa and Middle East	Lower middle income	14.75	14.53	11.27	11.15	0.12	-23.59	-23.26	0.33	-43.54	119.1	161.2
Tajikistan	Caucasus and Central Asia	Lower middle income	19.36	18.99	14.18	13.98	0.20	-26.76	-26.38	0.38	-31.70	64.3	69.0
Tanzania	Sub-Saharan Africa	Low income	32.68	31.65	22.94	22.42	0.52	-29.8	-29.16	0.64	-41.09	102.2	121.8
Thailand	Southeastern Asia and Oceania	Upper middle income	7.74	7.68	5.00	4.97	0.03	-35.4	-35.29	0.11	-47.24	61	74.7
Timor-Leste	Southeastern Asia and Oceania	Lower middle income	27.01	26.30	18.10	17.78	0.32	-32.99	-32.40	0.59	-40.32	72.1	81.0
Togo	Sub-Saharan Africa	Low income	46.24	44.20	35.36	34.15	1.21	-23.53	-22.74	0.79	-25.82	128.1	132.1
Tonga	Southeastern Asia and Oceania	Upper middle income	9.60	9.51	8.93	8.85	0.08	-6.98	-6.94	0.04	-10.05	130.4	134.9
Trinidad and Tobago	Latin America and the Caribbean	High income:	17.94	17.62	11.24	11.11	0.13	-37.35	-36.95	0.40	-29.50	96	85.3
Tunisia	North Africa and Middle East	Upper middle income	15.01	14.78	9.25	9.16	0.09	-38.37	-38.02	0.35	-55.42	81.7	112.9
Turkey	North Africa and Middle East	Upper middle income	12.91	12.75	7.05	7.00	0.05	-45.39	-45.10	0.29	-64.62	64.4	99.4
Turkmenistan	Caucasus and Central Asia	Upper middle income	22.96	22.45	17.31	17.02	0.29	-24.61	-24.19	0.42	-25.74	76	77.1
Tuvalu	Southeastern Asia and Oceania	Upper middle income	18.05	17.73	13.22	13.04	0.18	-26.76	-26.45	0.31	-23.82	75.3	72.4
Uganda	Sub-Saharan Africa	Low income	30.25	29.36	21.48	21.02	0.46	-28.99	-28.41	0.58	-42.23	93.3	114.7
Ukraine	Developed region	Lower middle income	12.50	12.34	8.88	8.81	0.07	-28.96	-28.61	0.35	-50.85	111.7	161.5
United Arab Emirates	North Africa and Middle East	High income:	8.98	8.90	7.42	7.37	0.05	-17.37	-17.19	0.18	-37.39	160.6	212.0
United Kingdom	Developed region	High income: OECD	3.67	3.66	2.95	2.94	0.01	-19.62	-19.67	-0.05	-36.84	96.6	122.9
United States	Developed region	High income: OECD	3.12	3.11	2.96	2.95	0.01	-5.13	-5.14	-0.01	-21.74	67.8	82.2
Uruguay	Latin America and the Caribbean	High income	8.57	8.50	6.59	6.55	0.04	-23.1	-22.94	0.16	-39.02	102.3	129.0
Uzbekistan	Caucasus and Central Asia	Lower middle income	15.88	15.63	12.19	12.04	0.15	-23.24	-22.97	0.27	-29.42	55.2	60.0
Vanuatu	Southeastern Asia and Oceania	Lower middle income	14.37	14.17	14.10	13.90	0.20	-1.88	-1.91	-0.03	-4.36	118.3	121.3
Venezuela, RB	Latin America and the Caribbean	High income	9.03	8.95	7.18	7.13	0.05	-20.49	-20.34	0.15	-25.79	75.4	80.8

Vietnam	Southeastern Asia and Oceania	Lower middle income	15.25	15.02	10.25	10.15	0.10	-32.79	-32.42	0.37	-27.28	97.4	90.1
West Bank and Gaza	North Africa and Middle East	Lower middle income	9.72	9.62	7.54	7.48	0.06	-22.43	-22.25	0.18	-28.04	59.4	64.0
Yemen, Rep.	North Africa and Middle East	Lower middle income	38.01	36.62	29.87	29.00	0.87	-21.42	-20.81	0.61	-40.94	101.2	134.6
Zambia	Sub-Saharan Africa	Lower middle income	27.02	26.31	21.30	20.86	0.44	-21.17	-20.71	0.46	-37.67	78.5	99.3
Zimbabwe	Sub-Saharan Africa	Low income	20.35	19.94	21.04	20.61	0.43	3.39	3.36	-0.03	12.41	97.1	89.3

Notes: SLBR and STBR stand for still live and total birth rates, SB for the number stillborn, and NM and NMR for the number and rate of neonatal mortality. The STBR is from Blencowe et al. (2016), SLBR is derived by using the number stillborn from Blencowe et al. (2016) and number of live births as calculated by using the neonatal mortality number and rate from World Development Indicators. Niue (population 1,190 in July 2014 per CIA World Fact Book) for which Blencowe et al. (2016) give the rate but not the number stillborn is excluded from the above table. The neonatal mortality rate and numbers for Cook Islands were obtained from Child Mortality Estimates by the UN Inter-agency Group. Income groups are as defined by the World Bank
