# Supplementary material for: Stillbirths: how should its rate be reported, its disability-adjusted-life-years (DALY), and stillbirths adjusted life expectancy
Source: BMC Med Inform Decis Mak. 2019 Jul 16;19:133. doi: 10.1186/s12911-019-0850-8 (PMC6631739; doi:10.1186/s12911-019-0850-8)
Supplement: Supplementary file 3 — Life expectancy & stillbirths adjusted life expectancy, 2000 & 2015, and i) decrease in life expectancy due to stillbirths, and ii) DALY = YLL of stillbirths, 2015, by country; format. (PDF 451 kb) [file 12911_2019_850_MOESM3_ESM.pdf]

Additional file 3: Life expectancy & stillbirths adjusted life expectancy, 2000 & 2015, and i) decrease in life expectancy due to stillbirths, and ii) DALY = YLL of stillbirths, 2015, by country

	SLBR		2000		2015			DALY=YLL	% Δ 2000-2015		
**Country name**	**2000**	**2015**	**LE**	**SALE**	**LE**	**SALE**	**|SALE-LE|**	**2015**	**LE**	**SALE**	**|SALE-LE|**
Afghanistan	37.01	27.48	55.13	53.16	60.72	59.10	1.62	1646853	10.14	11.17	-17.77
Albania	5.79	4.00	74.27	73.84	78.01	77.70	0.31	13987	5.04	5.23	-27.91
Algeria	26.70	19.70	70.18	68.35	75.04	73.59	1.45	1383723	6.93	7.67	-20.77
Angola	35.49	28.08	45.2	43.65	52.67	51.23	1.44	1564366	16.53	17.37	-7.10
Antigua & Barbuda	8.77	7.00	73.4	72.76	76.1	75.57	0.53	823	3.68	3.86	-17.19
Argentina	7.37	4.60	73.76	73.22	76.33	75.98	0.35	266883	3.48	3.77	-35.19
Armenia	21.48	13.96	71.28	69.78	74.8	73.77	1.03	41935	4.94	5.72	-31.33
Australia	3.37	2.72	79.23	78.96	82.45	82.23	0.22	65707	4.06	4.14	-18.52
Austria	4.67	3.64	78.13	77.77	81.84	81.54	0.30	24724	4.75	4.85	-16.67
Azerbaijan	25.85	16.74	66.76	65.08	70.85	69.68	1.17	273378	6.13	7.07	-30.36
Bahamas, The	11.27	10.35	72.25	71.44	75.4	74.63	0.77	4434	4.36	4.47	-4.94
Bahrain	9.48	5.55	74.6	73.90	76.82	76.40	0.42	9113	2.98	3.38	-40.00
Bangladesh	44.22	26.02	65.35	62.58	72	70.17	1.83	5810578	10.18	12.13	-33.94
Barbados	9.28	8.59	73.22	72.55	75.66	75.02	0.64	2187	3.33	3.40	-4.48
Belarus	5.66	2.97	68.91	68.52	73.62	73.40	0.22	27694	6.84	7.12	-43.59
Belgium	3.55	3.02	77.72	77.45	81.29	81.05	0.24	30327	4.59	4.65	-11.11
Belize	11.29	9.84	68.42	67.66	70.19	69.51	0.68	5747	2.59	2.73	-10.53
Benin	37.46	31.24	55.19	53.20	59.72	57.91	1.81	678582	8.21	8.85	-9.05
Bhutan	27.59	16.19	60.65	59.02	69.83	68.72	1.11	13671	15.14	16.44	-31.90
Bolivia	18.06	13.04	60.69	59.61	68.74	67.86	0.88	215802	13.26	13.84	-18.52
Bosnia and Herze.	6.74	5.48	74.26	73.76	76.59	76.17	0.42	13094	3.14	3.27	-16.00
Botswana	17.84	15.46	48.69	47.84	64.49	63.51	0.98	56519	32.45	32.76	15.29
Brazil	12.24	8.65	70.04	69.19	74.68	74.04	0.64	2060055	6.62	7.01	-24.71
Brunei Darussalam	6.98	6.52	75.29	74.77	79.04	78.53	0.51	3914	4.98	5.03	-1.92
Bulgaria	8.54	5.77	71.66	71.05	74.47	74.04	0.43	25754	3.92	4.21	-29.51
Burkina Faso	30.25	21.70	50.35	48.87	58.93	57.68	1.25	858169	17.04	18.03	-15.54
Burundi	37.27	27.36	51.49	49.64	57.11	55.59	1.52	701167	10.91	11.99	-17.84
Cabo Verde	19.76	14.49	69.72	68.37	73.36	72.31	1.05	11464	5.22	5.76	-22.22
Cambodia	21.27	12.08	58.35	57.13	68.66	67.84	0.82	291967	17.67	18.75	-32.79
Cameroon	24.70	19.96	51.94	50.69	55.93	54.84	1.09	892654	7.68	8.19	-12.80
Canada	3.54	3.15	79.24	78.96	82.14	81.88	0.26	95958	3.66	3.70	-7.14
Central African Republic	38.80	35.59	44.6	42.93	51.42	49.65	1.77	289797	15.29	15.65	5.99
Chad	43.19	41.58	47.58	45.61	51.87	49.80	2.07	1250158	9.02	9.19	5.08
Chile	3.91	3.07	76.82	76.52	81.79	81.54	0.25	58429	6.47	6.56	-16.67
China	14.73	7.20	71.73	70.69	75.99	75.45	0.54	9182935	5.94	6.73	-48.08
Colombia	11.00	8.14	70.99	70.22	74.18	73.58	0.60	449250	4.49	4.78	-22.08
Comoros	35.69	31.51	59.44	57.39	63.55	61.61	1.94	50454	6.91	7.35	-5.37
Congo, Dem. Rep.	35.40	28.03	50	48.29	59.02	57.41	1.61	5042628	18.04	18.89	-5.85
Congo, Rep.	21.49	15.29	50.59	49.53	62.87	61.92	0.95	155262	24.27	25.02	-10.38
Costa Rica	6.14	6.02	77.42	76.95	79.59	79.11	0.48	32583	2.80	2.81	2.13
Cote d'Ivoire	32.87	27.42	46.7	45.21	51.92	50.53	1.39	1147856	11.18	11.77	-6.71
Croatia	3.61	1.99	72.81	72.55	77.28	77.13	0.15	5625	6.14	6.31	-42.31
Cuba	11.11	6.18	76.64	75.80	79.55	79.06	0.49	57452	3.80	4.30	-41.67
Cyprus	5.12	3.71	77.97	77.57	80.31	80.01	0.30	3840	3.00	3.15	-25.00
Czech Republic	3.31	2.51	74.97	74.72	79.47	79.27	0.20	18714	6.00	6.09	-20.00
Denmark	3.42	1.74	76.59	76.33	81.1	80.96	0.14	7241	5.89	6.07	-46.15
Djibouti	45.71	35.85	57.01	54.52	62.29	60.13	2.16	47425	9.26	10.29	-13.25
Dominican Republic	13.49	11.19	70.63	69.69	73.68	72.86	0.82	177783	4.32	4.55	-12.77
Ecuador	11.03	7.74	72.94	72.14	76.1	75.52	0.58	189981	4.33	4.69	-27.50
Egypt, Arab Rep.	18.31	12.38	68.59	67.36	71.32	70.45	0.87	2439171	3.98	4.59	-29.27
El Salvador	17.61	12.30	68.72	67.53	73	72.11	0.89	93265	6.23	6.78	-25.21
Equatorial Guinea	21.62	16.44	52.11	51.01	57.96	57.02	0.94	26739	11.23	11.78	-14.55
Eritrea	28.59	23.04	56.03	54.47	64.1	62.66	1.44	238677	14.40	15.04	-7.69
Estonia	4.25	2.67	70.42	70.12	77.13	76.92	0.21	2557	9.53	9.70	-30.00
Ethiopia	40.70	30.59	51.93	49.90	64.58	62.66	1.92	6026527	24.36	25.57	-5.42
Fiji	14.36	12.06	67.61	66.65	70.26	69.42	0.84	14425	3.92	4.16	-12.50
Finland	2.65	1.85	77.47	77.27	81.39	81.24	0.15	8684	5.06	5.14	-25.00
France	5.49	4.74	79.06	78.63	82.67	82.28	0.39	306094	4.57	4.64	-9.30
Gabon	17.15	14.15	59.34	58.34	64.89	63.98	0.91	46685	9.35	9.67	-9.00
Gambia, The	31.72	24.44	55.58	53.87	60.47	59.03	1.44	118387	8.80	9.58	-15.79
Georgia	18.95	11.36	71.62	70.29	74.82	73.98	0.84	47626	4.47	5.25	-36.84
Germany	2.67	2.43	77.93	77.72	81.09	80.89	0.20	138065	4.05	4.08	-4.76
Ghana	30.56	23.22	56.99	55.30	61.49	60.09	1.40	1249892	7.90	8.66	-17.16
Greece	4.74	3.57	77.89	77.52	81.59	81.30	0.29	30772	4.75	4.88	-21.62
Grenada	9.06	8.00	70.25	69.62	73.52	72.94	0.58	1128	4.65	4.77	-7.94

Guatemala	17.16	12.08	67.74	66.60	71.96	71.10	0.86	382859	6.23	6.76	-24.56
Guinea	27.83	21.59	51.24	49.85	59.19	57.94	1.25	568791	15.52	16.23	-10.07
Guinea-Bissau	55.43	38.10	51.45	48.75	55.47	53.43	2.04	131872	7.81	9.60	-24.44
Guyana	20.13	17.55	64.97	63.69	66.51	65.36	1.15	19586	2.37	2.62	-10.16
Haiti	29.43	25.56	57.68	56.03	63.07	61.50	1.57	403237	9.34	9.76	-4.85
Honduras	18.06	12.77	70.49	69.24	73.33	72.41	0.92	149738	4.03	4.58	-26.40
Hungary	4.19	3.68	71.25	70.95	75.96	75.68	0.28	24355	6.61	6.67	-6.67
Iceland	2.89	1.13	79.65	79.42	82.86	82.77	0.09	394	4.03	4.22	-60.87
India	34.46	23.57	62.63	60.54	68.35	66.78	1.57	39200456	9.13	10.31	-24.88
Indonesia	17.99	13.41	66.25	65.08	69.07	68.16	0.91	4995741	4.26	4.73	-22.22
Iran, Islamic Rep.	9.53	6.48	70.14	69.48	75.59	75.10	0.49	660659	7.77	8.09	-25.76
Iraq	19.99	15.78	69.18	67.82	69.59	68.51	1.08	1314666	0.59	1.02	-20.59
Ireland	4.68	2.73	76.54	76.18	81.5	81.28	0.22	14740	6.48	6.69	-38.89
Israel	4.82	4.21	78.95	78.57	82.05	81.71	0.34	59606	3.93	4.00	-10.53
Italy	3.98	3.35	79.78	79.46	83.49	83.21	0.28	133048	4.65	4.72	-12.50
Jamaica	21.39	19.15	72.31	70.80	75.8	74.38	1.42	56695	4.83	5.06	-5.96
Japan	3.07	2.08	81.08	80.83	83.84	83.67	0.17	177905	3.40	3.51	-32.00
Jordan	13.14	10.60	71.78	70.85	74.2	73.42	0.78	160863	3.37	3.63	-16.13
Kazakhstan	11.18	6.52	65.52	64.80	72	71.53	0.47	184083	9.89	10.39	-34.72
Kenya	27.16	23.02	50.79	49.45	62.13	60.73	1.40	2119678	22.33	22.81	4.48
Kiribati	18.88	16.63	63.95	62.76	66.15	65.07	1.08	3047	3.44	3.68	-9.24
Korea, Dem. P. Rep	19.80	13.69	64.98	63.72	70.34	69.39	0.95	358782	8.25	8.90	-24.60
Korea, Rep.	2.76	2.14	75.84	75.63	82.16	81.98	0.18	82490	8.33	8.40	-14.29
Kuwait	6.65	5.10	73.31	72.83	74.7	74.32	0.38	26392	1.90	2.05	-20.83
Kyrgyz Republic	13.95	10.33	68.56	67.62	70.65	69.93	0.72	124522	3.05	3.42	-23.40
Lao PDR	32.68	24.30	58.88	57.02	66.54	64.96	1.58	272714	13.01	13.92	-15.05
Latvia	5.31	3.61	70.31	69.94	74.12	73.85	0.27	4265	5.42	5.59	-27.03
Lebanon	14.14	9.97	74.43	73.39	79.63	78.84	0.79	80781	6.99	7.43	-24.04
Lesotho	24.77	19.91	47.18	46.04	49.96	48.98	0.98	61136	5.89	6.39	-14.04
Liberia	32.51	21.88	52.41	50.76	61.16	59.85	1.31	197840	16.70	17.91	-20.61
Libya	12.64	8.87	70.57	69.69	71.83	71.20	0.63	78363	1.79	2.17	-28.41
Lithuania	5.82	3.24	72.02	71.60	75.12	74.88	0.24	7782	4.30	4.58	-42.86
Luxembourg	3.88	2.85	77.87	77.57	82.23	82.00	0.23	1533	5.60	5.71	-23.33
Macedonia, FYR	10.91	7.72	73.24	72.45	75.5	74.92	0.58	14379	3.09	3.41	-26.58
Madagascar	21.96	18.54	58.47	57.21	65.48	64.29	1.19	975501	11.99	12.38	-5.56
Malawi	29.57	22.30	44.08	42.81	63.8	62.41	1.39	873074	44.74	45.78	9.45
Malaysia	8.05	5.88	72.87	72.29	74.88	74.44	0.44	242587	2.76	2.97	-24.14
Maldives	19.56	7.87	70.06	68.72	76.98	76.38	0.60	4703	9.88	11.15	-55.22
Mali	45.98	33.63	48.9	46.75	58.46	56.56	1.90	1374120	19.55	20.98	-11.63
Malta	4.98	3.58	78.2	77.81	81.95	81.66	0.29	1080	4.80	4.95	-25.64
Marshall Islands	17.60	15.25	65.24	64.11	73.0	71.90	1.10	1486	11.89	12.15	-2.65
Mauritania	33.60	27.82	60.02	58.07	63.2	61.49	1.71	223877	5.30	5.89	-12.31
Mauritius	13.31	9.60	71.66	70.72	74.35	73.64	0.71	10049	3.75	4.13	-24.47
Mexico	7.03	5.52	74.3	73.78	76.92	76.50	0.42	996849	3.53	3.69	-19.23
Micronesia, Fed. Sts.	21.11	18.39	67.28	65.89	69.23	67.98	1.25	3104	2.90	3.17	-10.07
Moldova	10.44	7.93	66.89	66.20	71.63	71.07	0.56	24171	7.09	7.36	-18.84
Mongolia	12.58	7.34	62.91	62.13	69.82	69.31	0.51	36183	10.98	11.56	-34.62
Montenegro	6.00	3.99	73.18	72.74	76.34	76.04	0.30	2093	4.32	4.54	-31.82
Morocco	34.99	25.13	68.5	66.18	74.29	72.47	1.82	1308787	8.45	9.50	-21.55
Mozambique	27.85	19.50	48.69	47.37	55.37	54.31	1.06	1124273	13.72	14.65	-19.70
Myanmar	30.69	20.36	62.08	60.23	66.04	64.72	1.32	1208919	6.38	7.45	-28.65
Namibia	13.42	11.38	55.12	54.39	64.92	64.19	0.73	54461	17.78	18.02	0.00
Nepal	28.77	18.72	62.33	60.59	69.97	68.68	1.29	708360	12.26	13.35	-25.86
Netherlands	5.31	1.83	77.99	77.58	81.71	81.56	0.15	27000	4.77	5.13	-63.41
New Zealand	3.46	2.26	78.64	78.37	81.46	81.28	0.18	10723	3.59	3.71	-33.33
Nicaragua	11.02	7.50	69.66	68.90	75.1	74.54	0.56	67051	7.81	8.19	-26.32
Niger	40.62	38.07	50.7	48.72	61.97	59.70	2.27	2155744	22.23	22.54	14.65
Nigeria	55.17	44.81	46.62	44.18	53.05	50.77	2.28	15912954	13.79	14.92	-6.56
Norway	3.72	2.19	78.63	78.34	82.1	81.92	0.18	10980	4.41	4.57	-37.93
Oman	9.99	8.51	72.36	71.64	77.32	76.67	0.65	52788	6.85	7.02	-9.72
Pakistan	56.35	45.09	62.77	59.42	66.38	63.52	2.86	15238844	5.75	6.90	-14.63
Palau	10.95	9.01	70.49	69.73	73.0	72.35	0.65	183	3.56	3.76	-14.47
Panama	8.77	6.18	75.1	74.45	77.77	77.29	0.48	36066	3.56	3.81	-26.15
Papua New Guinea	19.21	16.17	58.8	57.69	62.78	61.78	1.00	208629	6.77	7.09	-9.91
Paraguay	19.19	13.57	70.07	68.75	73.03	72.05	0.98	132496	4.22	4.80	-25.76
Peru	13.92	9.03	70.51	69.54	74.78	74.11	0.67	417548	6.06	6.57	-30.93
Philippines	14.53	10.99	66.68	65.73	68.41	67.67	0.74	1743333	2.59	2.95	-22.11
Poland	4.69	2.35	73.75	73.41	78.2	78.02	0.18	65160	6.03	6.28	-47.06
Portugal	3.70	2.18	76.31	76.03	81.52	81.34	0.18	15060	6.83	6.98	-35.71
Qatar	7.58	5.75	76.5	75.92	78.76	78.31	0.45	12904	2.95	3.15	-22.41
Romania	6.80	4.15	71.16	70.68	74.96	74.65	0.31	51582	5.34	5.62	-35.42
Russian Federation	7.75	4.49	65.48	64.98	70.91	70.59	0.32	623961	8.29	8.63	-36.00
Rwanda	27.82	17.59	48.19	46.89	64.52	63.40	1.12	376154	33.89	35.21	-13.85

Samoa	12.10	11.05	69.49	68.66	73.76	72.95	0.81	3672	6.14	6.25	-2.41
San Marino	4.90	2.60	80.62	80.23	83.3	83.08	0.22	85	3.32	3.55	-43.59
Sao Tome & Principe	24.88	16.47	63.29	61.75	66.51	65.43	1.08	6835	5.09	5.96	-29.87
Saudi Arabia	19.22	14.13	72.53	71.16	74.49	73.45	1.04	641306	2.70	3.22	-24.09
Senegal	35.89	25.09	57.77	55.77	66.8	65.17	1.63	942937	15.63	16.85	-18.50
Serbia	7.84	5.98	71.58	71.02	75.49	75.04	0.45	41720	5.46	5.66	-19.64
Seychelles	11.32	9.83	72.4	71.59	73.23	72.52	0.71	1213	1.15	1.30	-12.35
Sierra Leone	40.71	24.96	38.69	37.18	51.31	50.06	1.25	272876	32.62	34.64	-17.22
Singapore	3.53	2.58	77.95	77.68	82.6	82.39	0.21	11200	5.97	6.06	-22.22
Slovak Republic	3.91	2.94	73.05	72.77	77.21	76.98	0.23	12452	5.69	5.79	-17.86
Slovenia	2.70	2.85	75.41	75.21	81.08	80.85	0.23	5038	7.52	7.50	15.00
Solomon Islands	20.04	17.91	62.84	61.61	68.15	66.95	1.20	20084	8.45	8.67	-2.44
Somalia	40.82	36.80	50.88	48.88	55.69	53.71	1.98	880191	9.45	9.88	-1.00
South Africa	22.94	17.76	55.84	54.59	57.44	56.44	1.00	1014226	2.87	3.39	-20.00
South Sudan	37.28	30.98	49.22	47.45	56.11	54.42	1.69	727065	14.00	14.69	-4.52
Spain	3.31	2.88	78.97	78.71	83.38	83.14	0.24	89074	5.58	5.63	-7.69
Sri Lanka	7.60	4.91	71.11	70.57	74.95	74.58	0.37	115405	5.40	5.68	-31.48
St. Lucia	13.18	12.16	71.42	70.49	75.18	74.28	0.90	2552	5.26	5.38	-3.23
St Vincent & the Grenadines	12.57	10.90	70.58	69.70	73.05	72.26	0.79	1285	3.50	3.67	-10.23
Sudan	30.09	24.99	57.97	56.28	63.71	62.16	1.55	1995625	9.90	10.45	-8.28
Suriname	24.64	19.82	67.93	66.30	71.29	69.90	1.39	13607	4.95	5.43	-14.72
Swaziland	16.20	12.50	48.66	47.88	48.87	48.27	0.60	21991	0.43	0.81	-23.08
Sweden	3.81	2.86	79.64	79.34	82.55	82.31	0.24	27100	3.65	3.74	-20.00
Switzerland	3.25	2.82	79.68	79.42	83.2	82.97	0.23	19685	4.42	4.47	-11.54
Syrian Arab Republic	14.75	11.27	72.72	71.66	70.09	69.31	0.78	330832	-3.62	-3.28	-26.42
Tajikistan	19.36	14.18	63.7	62.49	69.77	68.79	0.98	262004	9.53	10.08	-19.01
Tanzania	32.68	22.94	50.47	48.87	65.49	64.02	1.47	3009700	29.76	31.00	-8.13
Thailand	7.74	5.00	70.63	70.09	74.6	74.23	0.37	274082	5.62	5.91	-31.48
Timor-Leste	27.01	18.10	59.35	57.79	68.53	67.31	1.22	63429	15.47	16.47	-21.79
Togo	46.24	35.36	53.47	51.11	60.12	58.07	2.05	512578	12.44	13.62	-13.14
Tonga	9.60	8.93	70.76	70.09	72.94	72.29	0.65	1670	3.08	3.14	-2.99
Trinidad and Tobago	17.94	11.24	68.6	67.39	70.56	69.78	0.78	14743	2.86	3.55	-35.54
Tunisia	15.01	9.25	73.26	72.18	74.98	74.29	0.69	145185	2.35	2.92	-36.11
Turkey	12.91	7.05	70	69.11	75.43	74.90	0.53	745884	7.76	8.38	-40.45
Turkmenistan	22.96	17.31	63.9	62.47	65.74	64.62	1.12	128762	2.88	3.44	-21.68
Uganda	30.25	21.48	46.42	45.06	59.18	57.94	1.24	1968738	27.49	28.58	-8.82
Ukraine	12.50	8.88	67.86	67.02	71.19	70.56	0.63	272536	4.91	5.28	-25.00
United Arab Emirates	8.98	7.42	74.45	73.79	77.54	76.97	0.57	55165	4.15	4.31	-13.64
United Kingdom	3.67	2.95	77.74	77.46	81.6	81.36	0.24	182057	4.97	5.03	-14.29
United States	3.12	2.96	76.64	76.40	78.74	78.51	0.23	875684	2.74	2.76	-4.17
Uruguay	8.57	6.59	74.69	74.06	77.14	76.63	0.51	24562	3.28	3.47	-19.05
Uzbekistan	15.88	12.19	66.94	65.89	68.45	67.63	0.82	550803	2.26	2.64	-21.90
Vanuatu	14.37	14.10	67.56	66.60	72.16	71.16	1.00	6797	6.81	6.85	4.17
Venezuela, RB	9.03	7.18	72.35	71.70	74.41	73.88	0.53	318246	2.85	3.04	-18.46
Vietnam	15.25	10.25	73.15	72.05	75.78	75.01	0.77	1212550	3.60	4.11	-30.00
West Bank and Gaza	9.72	7.54	70.76	70.08	73.07	72.52	0.55	82653	3.26	3.48	-19.12
Yemen, Rep.	38.01	29.87	60.46	58.25	64.03	62.17	1.86	1528448	5.90	6.73	-15.84
Zambia	27.02	21.30	43.46	42.32	60.79	59.52	1.27	781240	39.88	40.64	11.40
Zimbabwe	20.35	21.04	41.69	40.86	59.16	57.94	1.22	664926	41.90	41.80	46.99

Notes: LE and SALE stand for traditional life expectancy and stillbirths adjusted life expectancy, respectively. Out of 194 countries in App. 2, life expectancy data are unavailable for Andorra, Cook Islands, Dominica, Monaco, Nauru, St. Kitts and Nevis, and Tuvalu. Traditional Life expectancy data is from World Development Indicators (WDI). WDI does not have 2015 life expectancy numbers for Marshall Islands and Palau. These numbers as estimated by U.S. Census Bureau: International Database are used. SLBR numbers are from App. 2. |SALE-LE| measures decrease in life expectancy due to considering stillbirths. The last column gives its % change.
